# Supplementary material for: Getting More Out of Biomedical Documents with GATE's Full Lifecycle Open Source Text Analytics
Source: PLoS Comput Biol. 2013 Feb 7;9(2):e1002854. doi: 10.1371/journal.pcbi.1002854 (PMC3567135; doi:10.1371/journal.pcbi.1002854)
Supplement: Dataset S1 — GATE software. Dataset S1 bundled with this paper contains a distribution of GATE (or see http://gate.ac.uk/download/). (TGZ) [file pcbi.1002854.s001.tgz › plos-gate/plugins/Tagger_MetaMap/doc/info/olteanu/utils/TextNormalizer.html]

TextNormalizer


---


|  |  |  |
| --- | --- | --- |
| **Overview** \| **Package** \| **Class** \| **Use** \| **Tree** \| **Deprecated** \| **Index** \| **Help** | |  |
| PREV CLASS   **NEXT CLASS** | **FRAMES**    **NO FRAMES**     **All Classes** |  |
| SUMMARY: NESTED \| FIELD \| CONSTR \| METHOD | DETAIL: FIELD \| CONSTR \| METHOD |  |


---


## info.olteanu.utils Class TextNormalizer

```
java.lang.Object
  info.olteanu.utils.TextNormalizer
```

---

``` public class TextNormalizer extends java.lang.Object ```

---

| **Nested Class Summary** | |
| --- | --- |
| `static class` | `TextNormalizer.Java5Normalizer` |
| `static class` | `TextNormalizer.Java6Normalizer` |


**Constructor Summary**

`TextNormalizer()`


| **Method Summary** | |
| --- | --- |
| `static StringFilter` | `getNormalizationStringFilter()` |

**Methods inherited from class java.lang.Object**

`clone, equals, finalize, getClass, hashCode, notify, notifyAll, toString, wait, wait, wait`

**Constructor Detail**

### TextNormalizer

```
public TextNormalizer()
```


**Method Detail**

### getNormalizationStringFilter

```
public static StringFilter getNormalizationStringFilter()
                                                 throws java.lang.ClassNotFoundException
```

:   **Throws:**: `java.lang.ClassNotFoundException`


---


|  |  |  |
| --- | --- | --- |
| **Overview** \| **Package** \| **Class** \| **Use** \| **Tree** \| **Deprecated** \| **Index** \| **Help** | |  |
| PREV CLASS   **NEXT CLASS** | **FRAMES**    **NO FRAMES**     **All Classes** |  |
| SUMMARY: NESTED \| FIELD \| CONSTR \| METHOD | DETAIL: FIELD \| CONSTR \| METHOD |  |


---
